# The pan-tropical age distribution of regenerating tropical moist forest

**DOI:** 10.1038/s41559-025-02721-8

**Published:** 2025-05-20

**Authors:** Christopher G. Bousfield, David P. Edwards

**Affiliations:** 1 Department of Plant Sciences and Centre for Global Wood Security, University of Cambridge, Cambridge, UK; 2 Conservation Research Institute, University of Cambridge, Cambridge, UK; 3 Ecology and Evolutionary Biology, School of Biosciences, University of Sheffield, Sheffield, UK

**Keywords:** Restoration ecology, Tropical ecology, Forest ecology

## Abstract

Natural forest regeneration in the tropics is a key element of restoration pledges. Protecting older regenerating forests that already hold substantial carbon and biodiversity value, while promoting natural regeneration in young secondary forests in regions where forests are likely to persist long term, is vital for effective forest restoration. Key questions therefore include understanding the age distribution of naturally regenerating forests pan-tropically and which environmental or socio-economic conditions predict increased longevity in regenerating forests. Here, using a time series of forest cover data (1990–2023) to map the age of regenerating tropical moist forests, we identify 51 Mha of regenerating tropical moist forest, of which >50% is ≤5 years old and under high deforestation pressure, whereas only 6% (3 Mha) is ≥20 years old and located predominantly in the tropical Americas. Location and forest characteristics in the surrounding landscape best predict the age of regenerating forests, with older forests located in areas with high forest integrity and extent, and low forest loss. Realizing the environmental and social values of naturally regenerating forests requires urgent financial, political and societal mechanisms to facilitate the long-term persistence of restoration.

## Abstract (web summary)

Natural forest regeneration is key to biodiversity conservation and climate change mitigation. Here the authors present a high-resolution time-series map of the age of regenerating tropical moist forests and find that most are ≤5 years old, with forest characteristics and geography explaining patterns better than climate or human pressure.

## Main

Large-scale restoration of forest ecosystems has immense capacity to sequester globally important amounts of carbon to combat climate change^1^, create millions of hectares of habitat to safeguard biodiversity^2^ and contribute towards multiple Sustainable Development Goals^3^. Many global initiatives promote the restoration of degraded ecosystems, including the 2021–2030 United Nations Decade on Ecosystem Restoration^4^, Target 2 of the Global Biodiversity Framework to restore 30% of all degraded ecosystems by 2030^5^ and the Bonn Challenge to bring 350 million hectares (Mha) of degraded landscapes under restoration by 2030^6^. Such international commitments have created a critical opportunity to restore degraded ecosystems globally.

Forest restoration in the tropics is a key element of global restoration strategies, as it offers the largest benefits for both carbon and biodiversity at comparatively low economic cost^2,7,8^. Natural forest regeneration—naturally occurring regrowth on previously deforested and degraded land, sometimes assisted by direct planting of native seedlings—accounts for 34% (64 Mha) of all tropical restoration pledges^3^. The remainder consists of commercial tree plantations (for example, for timber) or agroforestry^3^, yet national-level pledges are often ambitious, untracked and can even exceed the total area available for restoration in many countries^9^.

Naturally regenerating forests store more carbon, support more biodiversity and provide greater ecosystem services (that is, water provision and soil erosion control) than plantations^10^. Recovery of forest biomass and structure in naturally regenerating tropical forests is rapid, up to 100 times faster than in slower-growing regions (for example, boreal forest)^7^, with carbon stocks capable of reaching 85% of old-growth values in just 20 years, and 90% of old-growth values in 66 years on average^11^. Biodiversity of most plant and animal taxa can also recover rapidly, with up to 80% of old-growth species richness levels recovered in 20 years, and full recovery after 50 years^12–15^ (though full recovery of species composition can take centuries)^12^. This rapid recovery of biodiversity, carbon and forest function makes natural regeneration of tropical forests one of the most effective and cost-effective forms of ecosystem restoration.

Natural forest regeneration is feasible across an estimated 215 Mha of deforested tropical land^16^, indicating widespread opportunities to restore natural forest ecosystems across the tropics. Restoration would be most effective if these initiatives ensure the longevity of the new forests they promote or target the protection and further recovery of existing older naturally regenerating forests that are in danger of being deforested^17^. But regenerating forests are frequently at risk of clearance. In the Amazon, for example, almost half of naturally regenerating forests are ≤5 years old^17,18^ and subject to increased clearance^19^, and the probability of persistence is strongly linked to surrounding tree cover and proximity to existing forests in southern Costa Rica (CRI) and the Brazilian Atlantic Forest^20,21^. However, the age distribution of regenerating forests, and conditions that promote their persistence across the tropics remains unclear.

A key question is understanding the age and spatial distribution of regenerating tropical forests and the environmental and social conditions that support recovery. This would allow for targeted conservation of older regenerating forests and focused restoration efforts in areas that are more likely to support longer-term forest recovery. Here, we develop a 30 m-resolution mapped time series of the age of regenerating tropical moist forest (TMF) growing on previously deforested land using the latest version of a remotely sensed land-use change dataset covering the period 1990 and 2023^22^. We use this to answer the following questions: (1) where are the oldest regenerating TMFs, (2) which regions and nations are most important for harbouring older regenerating forests? and (3) what socio-environmental landscape characteristics best predict regenerating forest age?

## Results

### Age distribution of regenerating forests across the tropics

**Fig. 1:**
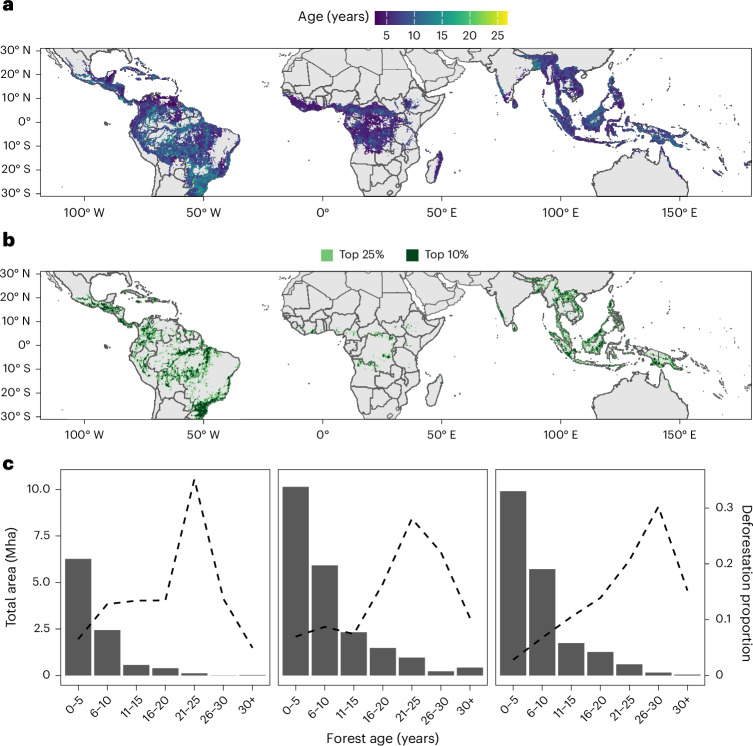
Age distribution of regenerating TMF. **a**, The mean age of regenerating TMF per 30 km grid cell. **b**, The hotspots of total area of older (≥20 years) regenerating TMF within 30-km cells. Cells in the 75th percentile for total area of old regenerating TMF are coloured in light green and cells in the 90th percentile are coloured in dark green. **c**, The area distribution (Mha) of regenerating TMFs among age classes in the tropical Americas (left), Africa (middle) and Asia–Pacific (right). The dashed line represents the proportion of all deforestation (1990–2023) that occurred during the corresponding time period (for example, height of the line at 6–10 years represents the proportion of deforestation between 1990 and 2023 that occurred 6–10 years ago).

We used a time series (1990–2023) of the annual extent of TMF^22^ (defined as a closed forest with >90% canopy cover located in the humid tropics, not including tropical dry forests) to map and age regenerating forests across the tropics at 30 m resolution. In 2023, there was 51 Mha of regenerating TMF, with 21.5 Mha found in the tropical Americas, followed by 19.4 Mha in Asia–Pacific and 9.8 Mha in Africa (Fig. 1). Just five countries—Brazil (BRA, 10.6 Mha), Indonesia (IDN, 7.2 Mha), Democratic Republic of Congo (COD, 3.3 Mha), Colombia (COL, 2.6 Mha) and Myanmar (MMR, 2.2 Mha)—account for over half (52%) of the world’s regenerating TMFs. Globally, regenerating TMF has a mean age of 7.6 ± 5.9 years (median age of 5 years) but demonstrates regional variation, with mean ages of 8.4 ± 6.7, 6.0 ± 4.2 and 7.4 ± 5.3 years (median ages 6, 5 and 5 years) in the Americas, Africa and Asia–Pacific, respectively.

Only 6% (3 Mha) of regenerating TMF is ≥20 years old. Of this ‘old’ regenerating forest, 63% (1.9 Mha) is located in the American tropics, 31% (0.9 Mha) in the Asian-Pacific tropics and only 7% (0.2 Mha) in the African tropics (Fig. 1b). Hotspots of old regenerating TMF extent are located predominantly in the Amazon Basin, Brazilian Atlantic Forest, Central America and Congo Basin as well as Borneo, Indo-Burma and southern Papua New Guinea (PNG). Many of these areas overlap with global biodiversity and conservation hotspots^23^, areas with the highest carbon accumulation potential in regenerating forests^7^, and restoration opportunity hotspots^24^, highlighting the important role that older regenerating TMFs could play in global conservation and restoration efforts.

By contrast, 52% (26.3 Mha) of regenerating TMF is 5 years old or younger, of which 39% (10.1 Mha), 24% (6.3 Mha) and 38% (9.9 Mha) is located in the Americas, Africa and Asia–Pacific, respectively (Fig. 1c; see Supplementary Figs. 1 and 2 for national-level distributions). As seen in Brazil^17,21^, this young forest is probably at high risk of conversion. In Costa Rica, where the relative extent of old regenerating forest was comparatively large, regional studies still suggest that >50% of naturally regenerating forests are cleared within 20 years of establishment^20^. Similarly, in the Brazilian Atlantic, a hotspot of older regenerating forests, the turnover of ‘ephemeral’ regenerating forests is high, with a mean age of just 7.9 years^21^, while 70% of cleared regenerating forest in the Amazon are 5 years old or younger^17^.

### Older regenerating forests are concentrated in the Americas

**Fig. 2:**
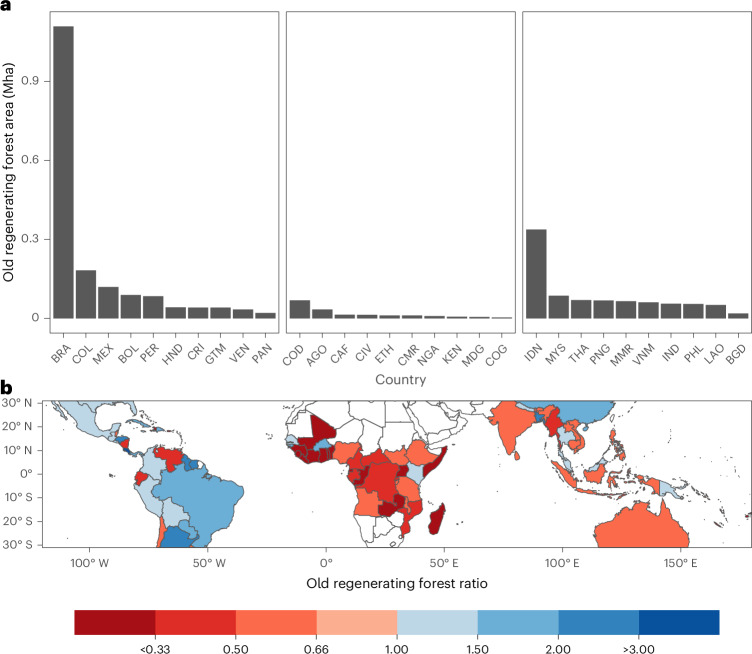
National-level distribution of older regenerating TMF. **a**, Total area (in Mha) of the ten countries with the largest extent of old (≥20 years) regenerating TMF by region (Americas, Africa and Asia–Pacific). **b**, The national-level ratio of the proportion of all pan-tropical old regenerating TMF compared to the proportion of all regenerating TMF of any age. A ratio of 1 means a country has the same share of all old regenerating forest as it does all regenerating forest of any age across the tropics (for example, 10% of all old regenerating forest and 10% of all regenerating forest). Blue colours represent ratios >1, whereby the country has more old regenerating forest than would be expected based on its share of all regenerating forest, red colours represent ratios <1, whereby the country has less old regenerating forest than would be expected. MEX, México; BOL, Bolivia; PER, Peru; GTM, Guatemala; VEN, Venezuela; PAN, Panama; AGO, Angola; CAF, Central African Republic; CIV Côte d'Ivoire; ETH, Ethiopia; CMR, Cameroon; NGA, Nigeria; KEN, Kenya; MDG, Madagascar; COG, Republic of the Congo; MYS, Malaysia; THA, Thailand; IND, India; PHL, Philippines.

At a national scale, Brazil accounts for 37% (1.1 Mha) of old regenerating TMF (≥20 years), and combined with Indonesia (0.3 Mha) and Colombia (0.2 Mha), represents >50% of global old regenerating TMF (Fig. 2a). Of the 30 countries with the largest extent of old regenerating forest, 14 are tropical American, 10 Asian–Pacific and only 6 African (Fig. 2a). Tropical American countries also account for a higher share of pan-tropical older regenerating TMF area than would be expected based on their pan-tropical share of regenerating forest area of all ages (Fig. 2b), holding 63% of old regenerating TMF in just 42% of the total regenerating TMF area. Conversely, Africa and Asia–Pacific hold less old regenerating forest than would be expected given their share of pan-tropical regenerating forest extent (Africa is composed of 6% of old regenerating forest on 19% of regenerating forest; Asia–Pacific is composed of 31% of old regenerating forest on 38% of regenerating forest). The drivers behind these continental-scale differences are unclear, but could be due to greater agricultural intensification^25^, increased shares of populations living in urban areas and declines of rural populations^26,27^ or forest transitions as a result of greater economic growth^28^. Similar deforestation trajectories across the regions since 1990 (Fig. 1c) suggest that the observed variance in regenerating forest age distributions is not simply a legacy of the timing of past deforestation events.

Of the 50 countries with regenerating TMF in the Americas, 21 have more old regenerating forest than would be expected based on their total regenerating forest extent, with Costa Rica, Honduras (HND) and Brazil supporting 3.7, 2.6 and 1.8 fold more old regenerating forest than expected, respectively (Fig. 2b). High extents of older regenerating TMFs in countries, such as Costa Rica, suggests national-level policies focusing on forest conservation (for example, the 1996 Forest Law) may impact the age distribution of regenerating forest extent^29^. By contrast, only 5 of 35 African countries have more old regenerating TMF than expected, with many West African countries (for example, Ghana and Sierra Leone) skewed towards younger forests and holding ten times less older-regenerating forest than expected, probably owing to the dominance of shifting cultivation in these countries and sub-Saharan Africa more broadly^30^ (Fig. 2b). Shifting cultivation itself is an important land-use system that occurs over 280 Mha worldwide^31^, and produces a mosaic of habitat capable of sustaining high species richness and carbon stocks^32^, especially when compared with intensive agriculture^33^. Tropical Asia–Pacific is more mixed, with 8 of 23 countries having more old regenerating tropical forest than expected, including Bangladesh (BGD) and Papua New Guinea with 2.5 and 1.3 times more old regenerating forest, respectively, while other countries such as Myanmar and Vietnam (VNM) have only half the expected old forest (Fig. 2b). Myanmar, Laos (LAO) and Vietnam are the three countries with the greatest extent of shifting cultivation in tropical Asia–Pacific^34^, which could explain their younger regenerating forest age distributions, while Papua New Guinea has larger remaining areas of undisturbed primary forest and less historical forest loss compared with other regions, such as Borneo^22^, perhaps leading to greater prevalence of older regenerating forest.

### Landscape forest characteristics best predict forest age

**Fig. 3:**
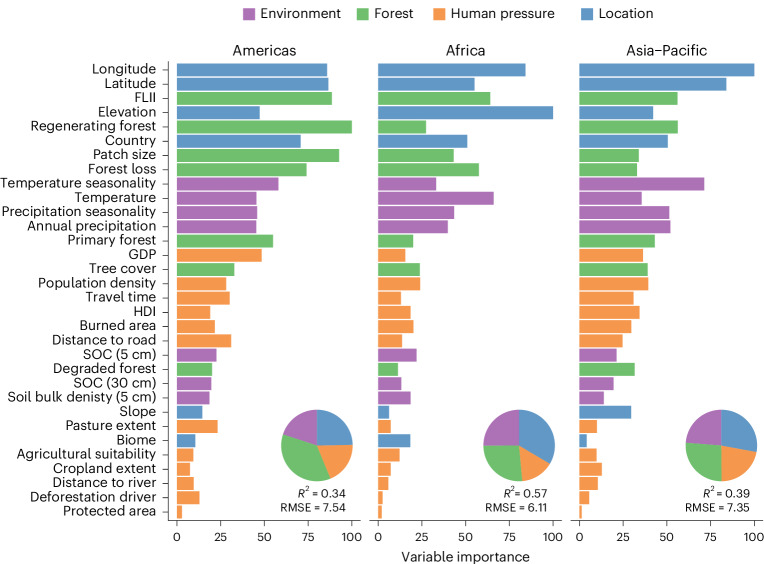
Variable importance scores for predicting regenerating TMF age. The variable importance scores from the random forest models for each region, with variables ordered by their total predictive power across all three regional models and coloured by type: environmental variables (purple), variables concerning the surrounding forest landscape (green), variables relating to levels of human pressure (orange) and variables relating to the location of the regenerating forest (blue). Insets: random forest model error (RMSE) and variance explained (*R*^2^) for each region. GDP, gross domestic product; HDI, human development; SOC, soil organic carbon.

**Fig. 4:**
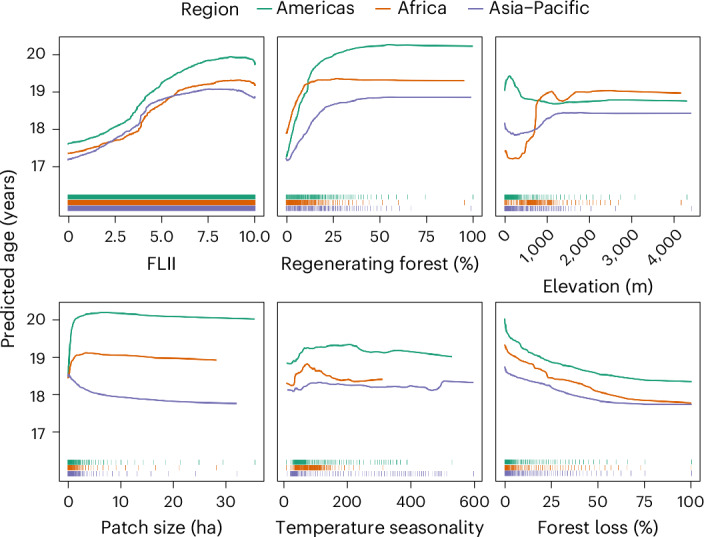
Partial effects of the six most important predictors of regenerating TMF age. The partial effect plots on regenerating forest age of the top six most important variables across the three models (excluding longitude, latitude and country) in order of importance (left to right). The regional-level partial effects are coloured by region: Americas (green), Africa (orange) and Asia–Pacific (purple). The distribution of data points for modelling partial effects in each region is shown at the bottom of each plot. Predicted age values represent predictions from the stratified sample of secondary forest points used to train the random forest models, and not the raw data, which was heavily skewed towards younger forests.

We used random forest models with 32 predictor variables (see Supplementary Table 1 for more detail), spanning factors related to forest, environment, location and human pressure, to determine which are most important for predicting regenerating TMF age at a 30 m resolution. Location variables, including longitude, latitude and country had high predictive power, indicating that older regenerating forests are spatially clustered across landscapes and regions, and that national-level policy and cultural practices can have strong impacts on the age distribution of regenerating forest^29^ (Fig. 3). Additionally, elevation was the most important predictor in Africa (but less so in the Americas and Asia–Pacific), where forests tended to be increasingly old as altitude increased to ~2,000 metres above sea level (Fig. 4), probably owing to the lower deforestation threat at higher elevations as land becomes increasingly marginal for agriculture^35^.

Forest landscape characteristics tended to be very important in predicting regenerating forest age, particularly in the Americas (Fig. 3; see Supplementary Fig. 3 for pan-tropical model). Landscapes with higher Forest Landscape Integrity Index (FLII) scores^36^, a larger extent of regenerating forests (especially in the Americas) and lower levels of forest loss tended to have older regenerating forests (Fig. 4; see Supplementary Fig. 4 for partial-effects of all variables). This supports regional studies in Costa Rica and Brazil, which found that nearby forest cover was one of the most important predictors of regenerating forest persistence^20,21^, while local forest density and distance to existing forests are important predictors of natural forest regeneration potential at the pan-tropical scale^16^. Older regenerating forests located in high forest density landscapes are also likely to be supporting elevated biodiversity given landscape forest extent is one of the most important predictors of biodiversity recovery in naturally regenerating forests^37^. Our results therefore indicate that restoration efforts focusing on natural regeneration should target areas with high surrounding forest landscape integrity to increase the chances of long-term restoration success.

Some environmental variables were also important in predicting the age of regenerating TMF, in particular temperature seasonality (Fig. 4), whereby predicted age increased as seasonality grew to intermediate levels before either plateauing (Americas) or dropping (Africa and Asia–Pacific) as seasonality became more extreme. Factors relating directly to forms of human pressure on the landscape (for example, deforestation drivers, agricultural suitability, cropland extent and protected area status) had little influence. This poor predictive power may be due to coarse-resolution mapping of some variables (for example, deforestation driver at 10 km^2^)^30^ rendering them unable to capture the substantial age variation within landscapes or because other predictors without readily available pantropical data (for example, land tenure security and land-use history)^38^ may have stronger influences over the longevity of regenerating forest. Furthermore, the high predictive power of variables, such as forest loss, regenerating forest extent and FLII, are indirectly reflective of human pressures. Natural regeneration potential is also highest in heavily forested landscapes^16^, and plays a vital role even where tree planting is used to initiate forest recovery^39^. Our results indicate that restoration efforts focusing on natural regeneration should target areas with high surrounding forest landscape integrity to increase the chances of long-term restoration success.

The greatest uncertainty in our study originates in the difficulty of accurately distinguishing naturally regenerating forest from tree plantations, which have expanded across the tropics at a similar rate to naturally regenerating forests^40^. To avoid confusion with oil palm, we used a pan-tropical dataset of smallholder and industrial oil palm plantations to mask out known oil palm plantations from our dataset^41^. We compared our data on regenerating TMFs with a recent global map of forest management^42^ and found minimal overlap with areas confidently identified as tree plantations or agroforestry (Supplementary Note 1 and Supplementary Table 2). In addition, the short rotation times of tropical tree plantations (for example, <10 years in *Eucalyptus* and *Acacia* plantations across much of the tropics)^43–45^ means that areas of old regenerating forests with detectable tree cover for ≥20 years are unlikely to have been misidentified tree plantations. We also compared our results with what is, to our knowledge, the only publicly available large dataset of regenerating forest age compiled for Brazil as part of the MapBiomas project^46,47^ (Supplementary Note 2 and Supplementary Fig. 5). Despite substantially different definitions of regenerating forest between the two datasets, we found moderate agreement between the datasets (Supplementary Fig. 5) in both regenerating forest area (*R*^2^ = 0.49) and age (*R*^2^ = 0.37). Additional analyses assessing the age distribution of regenerating forests at regional scales also found similar age profiles for regenerating forests across the Brazilian Amazon^48^ and Amazon Biome^17^.

## Discussion

Our study identifies 3 Mha of old regenerating TMF (≥20 years) located predominantly across the tropical Americas, typically in regions with high forest integrity and low deforestation rates, that will already hold substantial biodiversity and carbon value^11,12^ and should be priority targets for conservation and further restoration. However, we also find that the majority of naturally regenerating TMF is ≤5 years old, and probably caught in continuous cycles of farm abandonment, regeneration, deforestation and agricultural production, leaving them unable to accrue substantial carbon stocks or biodiversity in the long term. Using spatially explicit models of the carbon accumulation potential of naturally regenerating forests^7^, we estimate that protecting all 26 Mha of young regenerating TMF for the next 25 years would result in a potential above-ground sequestration of ~3 gigatons of carbon (GtC) by 2050. Ensuring the protection of naturally regenerating forests from re-clearance through financial, political and societal mechanisms should thus be key a target for the global restoration agenda to ensure long-term provision of forest ecosystem services^49^.

Carbon-financing schemes through Reducing Emissions from Deforestation and Forest Degradation (REDD+), offer a route towards funding the protection of regenerating forests, potentially creating a financially viable alternative to re-clearance for agricultural production^50^. Such schemes can also be used to promote assisted natural regeneration (ANR), which could enhance the recovery of carbon and biodiversity relative to passive regeneration^49^ in a cost-effective manner^51,52^ that also generates employment opportunities across the cycle of activities (for example, seed collection, nursery, land preparation, direct carbon payments to landowners and land-holding communities), thus incentivising the longer-term protection of these forests. However, methodologies to certify natural forest regeneration are lacking. Requirements for ten consistent years of forested or unforested land cover before an area qualifies for REDD+ or afforestation, reforestation and revegetation, and the premise that regenerating forests provide no additionality because they are already growing (despite their elevated deforestation risk^19^) prevents most young regenerating forests from qualifying for any form of carbon-financing scheme^53^. Under current regulations, regenerating forests therefore have limited economic value and cannot counter the often-marginal opportunity cost of conversion to agricultural land^21,50^. Unlocking this funding gap and prescribing economic value to regenerating forests will be vital for preventing ongoing future losses of ecologically valuable forest systems.

Effective regulatory protection for regenerating forests and community engagement in restoration and protection is also key. The combination of strict legislation in Brazil’s Forest Code and specific legal protections in the Brazilian Atlantic Forest that prevent clearance of regenerating forests >10 years old^54^ may be contributing towards the widespread natural regeneration occurring there^55^. Expanding these protections across the tropics through coherent and consistent legal frameworks^56,57^ could promote increased forest longevity. However, protections must be carefully designed to avoid perverse outcomes. Environmental service payments in Costa Rica economically favour the replacement of naturally regenerating forests with native tree plantations that have lower the environmental value^10,37,57^, while the 2008 Amazonian beef moratorium successfully slowed primary forest loss in Brazil but at the expense of naturally regenerating forests, which suffered a 280% increase in clearance rates^58^. Community participation and engagement in reforestation projects, as well as benefits sharing, is also critical in achieving socio-ecological benefits of restoration and long-term persistence^59,60^. Officially recognised indigenous territories have lower deforestation rates throughout the tropics^61^, while in the Brazilian Amazon collective property rights leads to larger, older regenerating forest extents^38^. Engaging communities in restoration efforts and improving the security of land-tenure could therefore increase the long-term success of naturally regenerating forests in tropical landscapes.

Addressing the underlying societal causes of deforestation in regenerating forests would ensure greater longevity and provision of ecosystem services where natural regeneration occurs. Where smallholder cultivation is leading to losses of regenerating forest, programmes to improve yields^62^ and diversify income streams for local communities (for example, through provision of timber or non-timber forest products or carbon-funded ANR)^63^ could reduce deforestation pressure. At the global scale, closing yield gaps and implementing economically efficient land-use decisions could increase agricultural production by 79–148%^64^, while shifts away from land-intensive diets relying heavily on ruminant meats could reduce land requirements by up to 70%, opening up some of the ~3.7 billion hectares used for livestock production^65^ to long-term natural regeneration. Expansion of naturally regenerating forests into abandoned crop and pastureland through natural colonisation and ANR^49^ could then be strategically targeted to maximise the environmental and social values of natural regeneration^2,7,16,57^.

Overall, this study highlights that despite the existence of pockets of long-term natural regeneration in the tropics, the potential restoration value of naturally regenerating tropical forests is currently not being realised. Alongside the protection of remaining old-growth forests, preventing the continued cyclical deforestation of naturally regenerating forests through economic investment, community engagement, effective regulation and societal shifts in diet will be crucial in realising the restoration potential of naturally regenerating forests. Only through these pathways will regenerating forests transition from ephemeral ecosystems^20,21^ into a key pillar of global restoration efforts.

## Methods

### Mapping regenerating TMF age

To create our map of tropical regenerating forest age, we used the TMF annual change dataset^22^. The TMF annual change product is a forest change dataset from the European Commision's Joint Research Centre that uses Landsat to track land cover change dynamics in humid TMFs (>90% canopy cover) at a resolution of 30 m between the years 1990 and 2023^22^. To create the map of regenerating TMF age in 2023, we first used the transition map and retained only pixels identified as ‘regrowth forest’, that is, pixels demonstrating naturally regrowing vegetation for 3 years or more after a deforestation event that resulted in a period of more than 2.5 years without detectable tree cover. After masking to retain only regenerating TMF pixels in the year 2023, we then stacked each year of the annual change data (1990–2023) together. Following a similar method to Heinrich et al.^66^ and Silva et al.^18^, and starting from 1990, for every consecutive year where regenerating forest was detected in a pixel, the forest age value of that pixel increased by 1. If a different land-use classification was detected on a pixel (for example, owing to a deforestation event), the regenerating forest age for that pixel was reset to 0. The age of the pixel remained 0 until the next year that regenerating forest was detected in the pixel, at which point the age counter would start again from one. This process was repeated cumulatively for the years 1990 through to 2023, with the final layer representing the age of regenerating TMF (that is, how many cumulative years that regenerating forest had been detected in that pixel) in the year 2023. The requirement for 3 years of forest cover before designation as a ‘forest regrowth’ pixel and the earliest year of the annual change dataset (1990) thus give our regenerating forest age values a possible range of 3–34 years old.

To remove any potential oil palm plantations classified as regenerating forest in the dataset, we masked out all pixels mapped as oil palm plantations in the map produced by Descals et al.^41^. We also tested our map of regenerating forest age against the map of regenerating vegetation age for Brazil from the MapBiomas project^46,47^ (Supplementary Note 2 and Supplementary Fig. 5) and the global map of forest management by Lesiv et al.^42^ (2022) (Supplementary Note 1 and Supplementary Table 2).

The forest aging process was carried out in Google Earth Engine^67^, with all subsequent spatial analysis conducted in RStudio version 4.4.4^68^. We used the ‘terra’^69^ package to aggregate age maps for visualisation, identify pan-tropical hotspots of older regenerating TMF (defined as ≥20 years old) and analyse regenerating forest extent and age profiles for each country on the basis of national boundaries from GADM^70^. Maps were plotted on the basis of national boundaries from ‘rnaturalearth’^71^. To estimate the carbon accumulation potential of protecting all young (≤5 years old) naturally regenerating TMF for the next 25 years, we extracted carbon accumulation values from spatially explicit estimates of annual aboveground carbon accumulation in naturally regenerating forests^7^ and multiplied these by 25.

### Identifying the best predictors of regenerating forest age

To identify the most important variables in predicting the age of regenerating TMF pixels, we used random forest regression models to predict regenerating forest age on the basis of a number of input variables. Random forest models employ an ensemble of decision trees for predictions, and were used since they are generally robust to interactions between predictors and complex nonlinear and non-monotonic relationships^72^. To ensure that the models were computationally tractable across a sensible timeframe, we took a random-stratified sample of points across forest ages. For each possible value of forest age between 3 and 34 years we took a random sample of 10,000 points. For each point we then identified values for a suite of 32 spatially explicit predictors that could influence regenerating forest age and deforestation pressure, covering locational factors (see Supplementary Fig. 6 for a model without spatial coordinates), landscape level forest characteristics, environmental conditions and human-related deforestation pressures (see the Supplementary Information for more information). We removed any variables that were highly correlated (*R* > 0.9). This process was repeated for each tropical region (Americas, Africa and Asia–Pacific), and we fit a separate random forest model for each region.

Modelling was performed using the ‘ranger’ implementation of random forests^73^ and trained using the ‘caret’ package^74^. We used 75% of the data for model training, and held 25% back for model testing. To ensure that we fit the most accurate model possible, we used ‘caret’ to tune important model hyperparameters to identify the most effective model. We tested a range of values for both ‘ntree’ (100–1500) and ‘mtry’ (3-29) under sevenfold cross validation, and selected the combination of ‘ntree’ and ‘mtry’ that delivered the highest level of accuracy in an acceptable timeframe for computation. The final model therefore used 500 trees and 18 ‘mtry’ values. We calculated *R*^2^ scores on the basis of the predictive accuracy of the model on the held-back test data. To estimate the importance of each variable in predicting regenerating forest age, we used the corrected impurity measure which estimates mean decrease in accuracy and is unbiased in terms of the number of categories and category frequencies^75,76^, and created partial dependence plots for each variable using the *Dalex* package^77^.

### Reporting summary

Further information on research design is available in the Nature Portfolio Reporting Summary linked to this article.

## Supplementary information

Supplementary Information Supplementary Figs. 1–6, Tables 1 and 2 and Notes 1 and 2.

Reporting Summary

## Data Availability

The underlying TMF dataset used in this study is freely accessible and available via EU Science Hub at https://forobs.jrc.ec.europa.eu/TMF/data. Additional data sources used for random forest modelling are also freely accessible and available for download in the links provided in the Supplementary Information. The final map of regenerating tropical moist forest age for the year 2023 is available via Zenodo at 10.5281/zenodo.15120870 (ref. ^78^).

## Code availability

The code used to generate the results are available via Zenodo at 10.5281/zenodo.15120870 (ref. ^78^).
