# Supplementary material for: The pan-tropical age distribution of regenerating tropical moist forest
Source: Nat Ecol Evol. 2025 May 20;9(7):1205–13. doi: 10.1038/s41559-025-02721-8 (PMC12240804; doi:10.1038/s41559-025-02721-8)
Supplement: Supplementary file 1 — Supplementary Figs. 1–6, Tables 1 and 2 and Notes 1 and 2. [file 41559_2025_2721_MOESM1_ESM.pdf]

nature ecology & evolution

Supplementary information https://doi.org/10.1038/s41559-025-02721-8

The pan-tropical age distribution of regenerating tropical moist forest

In the format provided by the authors and unedited

Supplementary Information - The pan-tropical age distribution of regenerating tropical moist forest

Christopher G. Bousfield[1,2,3*] & David P. Edwards[1,2]

[1]Department of Plant Sciences and Centre for Global Wood Security, University of Cambridge, Cambridge, UK

[2]Conservation Research Institute, University of Cambridge, Cambridge, UK

[3]Ecology and Evolutionary Biology, School of Biosciences, University of Sheffield, Sheffield, UK

*Corresponding author: cgb48@cam.ac.uk

Supplementary Table 1. Summary of covariates used in the random forest models to predict forest age

Variable	Group	Description
Longitude	Location	Longitudinal location
Latitude	Location	Latitudinal location
Country	Location	Country boundaries from GADM[69]
Elevation	Location	Elevation derived from NASADEM at 30m resolution - http://lpdaac.usgs.gov/products/nasadem_hgtv001/
Slope	Location	Angle of slope derived from NASADEM at 30m resolution - http://lpdaac.usgs.gov/products/nasadem_hgtv001/
Biome	Location	Biome as per Olson *et al*. https://www.worldwildlife.org/publications/terrestrial-ecoregions-of-the-world
Forest Landscape Integrity Index	Forest	The FLII integrates data on observed and inferred forest pressures and lost forest connectivity to generate a continuous index of forest integrity as determined by degree of anthropogenic modification, at 300m resolution[35]
Regenerating forest extent	Forest	Proportion of regenerating tropical moist forest pixels in the wider landscape at 1km resolution[21]
Patch Size	Forest	Total area of connected regenerating tropical moist forest pixels
Forest loss	Forest	Proportion of forest loss pixels in the wider landscape at 1km resolution[79]
Primary forest extent	Forest	Proportion of undisturbed forest pixels in the wider landscape at 1km resolution[21]
Tree Cover	Forest	Total tree cover pixels in the wider landscape at 1km resolution MOD44B Vegetation Continuous Fields V6 https://developers.google.com/earth-engine/datasets/catalog/MODIS_006_MOD44B
Degraded forest extent	Forest	Proportion of degraded forest pixels in the wider landscape at 1km resolution[21]
Temperature	Environment	Average monthly temperature (∘C) between 1970-2000 at 1km resolution- Worldclim https://www.worldclim.org/data/bioclim.html#google_vignette
Temperature Seasonality	Environment	Annual seasonality of temperature (defined as SD x 100) between 1970-2000 at 1km resolution - Worldclim https://www.worldclim.org/data/bioclim.html#google_vignette

Precipitation	Environment	Average monthly precipitation (mm) between 1970-2000 at 1km resolution - Worldclim https://www.worldclim.org/data/bioclim.html#google_vignette
Precipitation seasonality	Environment	Annual Seasonality of precipitation (defined as SD x 100) between 1970-2000 at 1km resolution - Worldclim https://www.worldclim.org/data/bioclim.html#google_vignette
Soil Organic Carbon (5cm)	Environment	Soil organic carbon stock in t/ha for 0-5 cm depth interval at 250m resolution - https://soilgrids.org
Soil Organic Carbon (30cm)	Environment	Soil organic carbon stock in t/ha for 0-30 cm depth interval at 250m resolution - https://soilgrids.org
Soil Bulk Density (5cm)	Environment	Bulk density (fine earth) in cg/cm^3 at 5cm depth at 250m resolution - https://soilgrids.org
Population density	Human Pressure	Mean population density (number of persons per square km) between the years 2000-2020 derived from GPWv4 at 1km resolution - https://sedac.ciesin.columbia.edu/data/collection/gpw-v4
Burned area	Human Pressure	Mean annual percentage burned between 2001-2020 derived from GFED5 at 0.25◦ resolution[80]
Travel time	Human Pressure	Travel time in minutes for the year 2015 to the nearest urban centre of ≥ 5000 people at 1km resolution[81]
Distance to road	Human Pressure	Distance in metres from the nearest OSM major road in the year 2016 at a 100m resolution - https://hub.worldpop.org/geodata/listing?id=31
GDP	Human Pressure	Gridded global datasets for GDP between 1990-2015 at 10km resolution[82]
Human development index	Human Pressure	Gridded global datasets for human development index 1990-2015 at 10km resolution[82]
Pasture extent	Human Pressure	Proportion of cells mapped as pasture land at 1km resolution[83]
Cropland extent	Human Pressure	Proportion of cells mapped as cropland by the ESA Worldcover 2021 10m product at 1km resolution[84]
Agricultural suitability	Human Pressure	Agricultural suitability score (0-100) for growing the world's 16 most important crops as calculated at 1km resolution by Zabel *et al.*[85]

Distance to river	Human Pressure	Distance in metres to nearest river with a catchment area of at least 10 km^2 or an average river flow of at least 0.1 m^3/sec, as per the HydroRIVERS database[86]
Deforestation driver	Human Pressure	Primary driver of forest loss between 2001-2023 at 10km resolution as predicted by Curtis *et al.* [29]
Protected Area	Human Pressure	Whether the pixel is located inside a designated Protected Area according to the World Database on Protected Areas as of 2022[87]

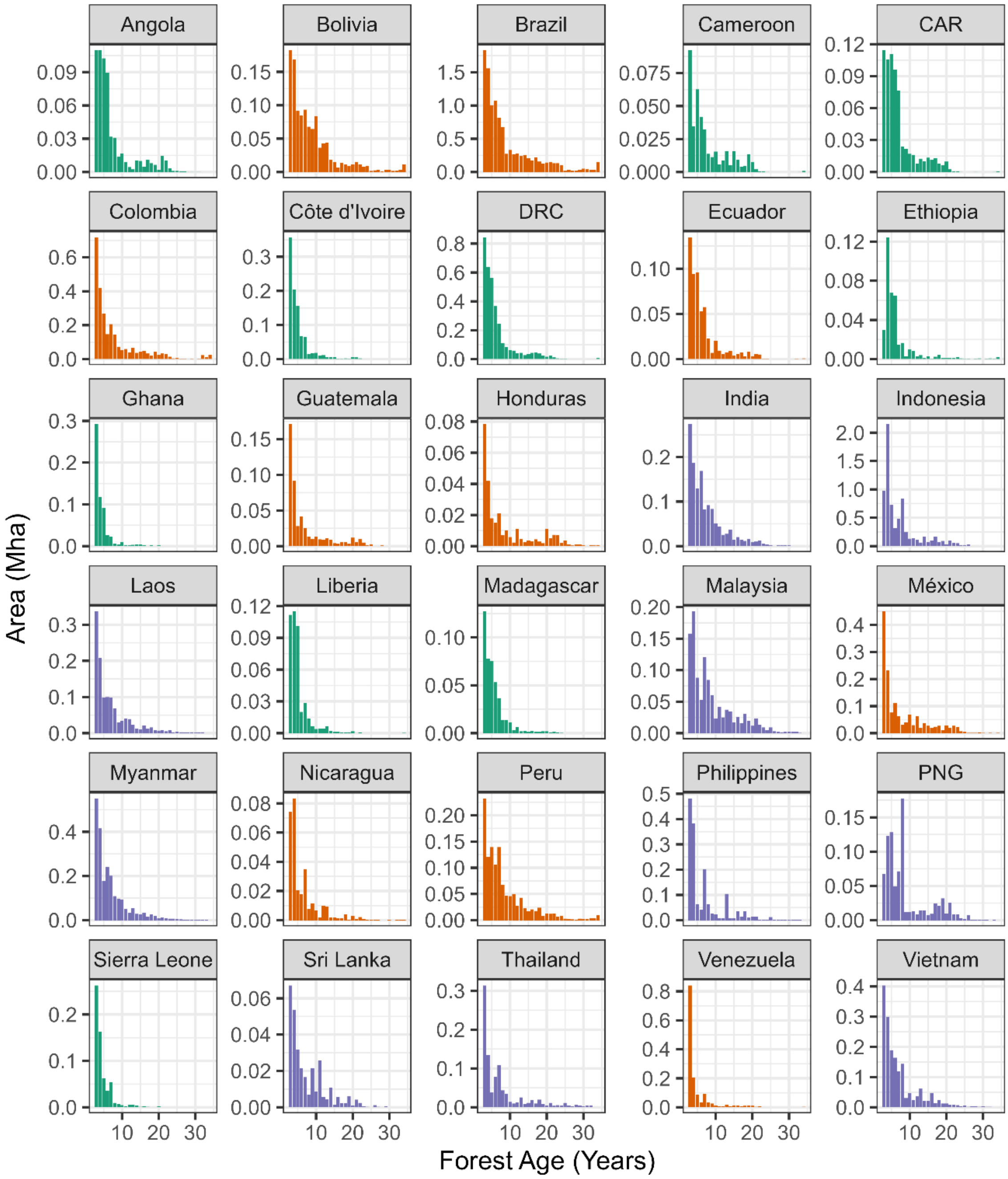

Supplementary Figure 1. National-level age distribution by area of regenerating tropical moist forest. Shown are the age distributions from the ten countries with the largest regenerating tropical moist forest extent for each region: Americas (green), Africa (orange) and Asia-Pacific (purple).

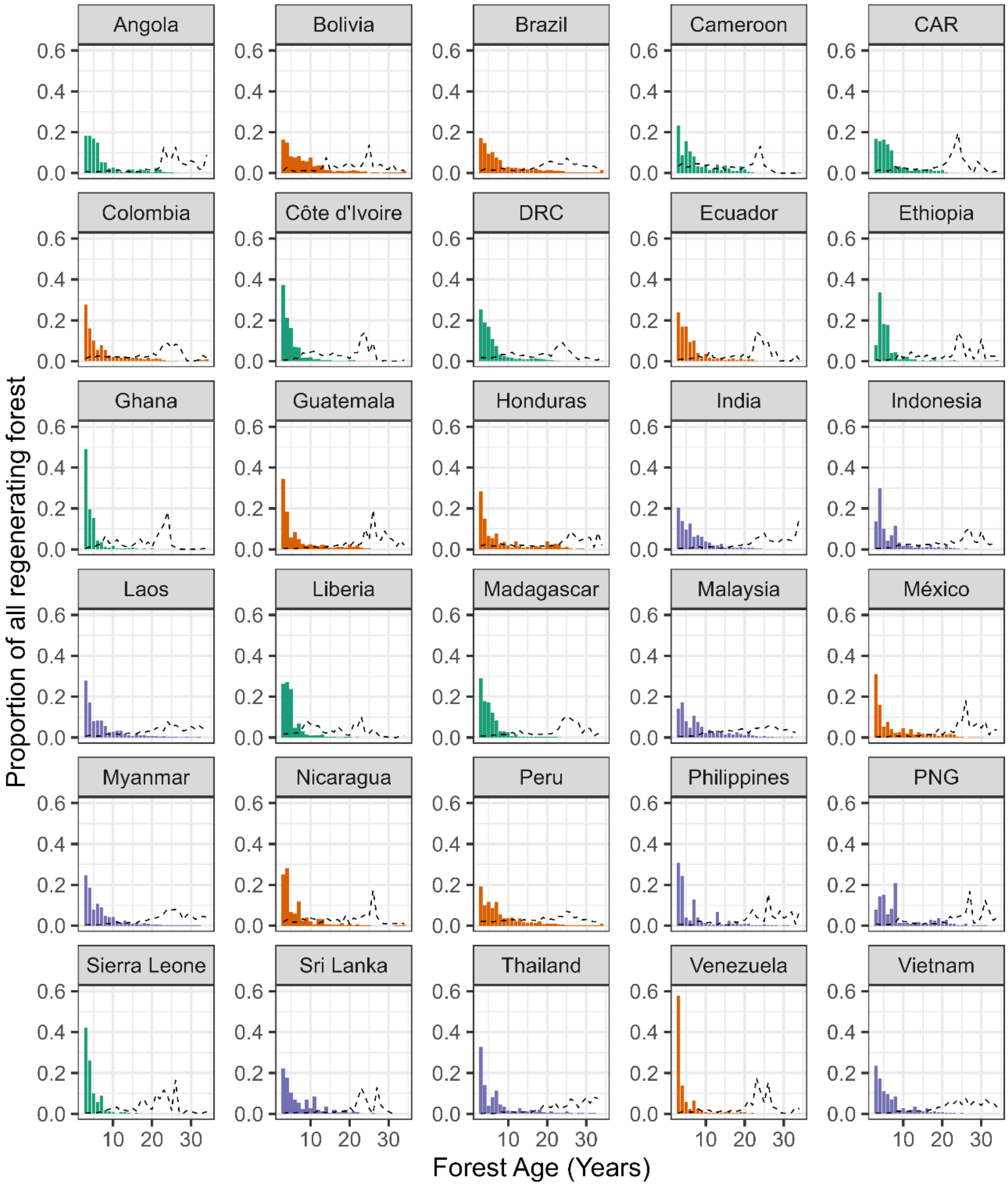

Supplementary Figure 2. National-level age distribution by proportion of regenerating tropical moist forest. Shown are the age distributions from the ten countries with the largest regenerating tropical moist forest extent for each region: Americas (green), Africa (orange) and Asia-Pacific (purple). Dashed line represents the proportion of all deforestation (1990-2023) that occurred during the corresponding time period (e.g. height of the line at 10 years represents the proportion of deforestation between 1990-2023 that occurred10 years ago).

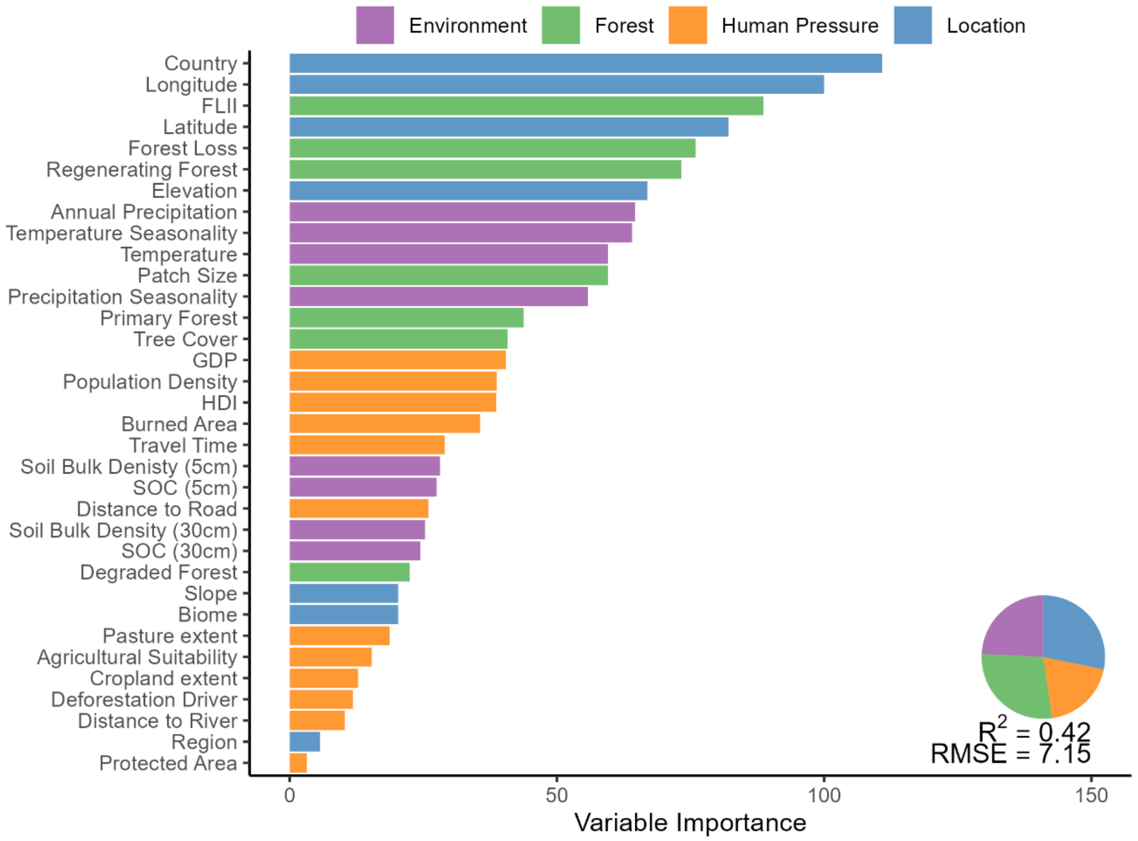

Supplementary Figure 3. Variable importance scores for predicting regenerating tropical moist forest age in one pan-tropical model. Shown are the variable importance scores from the random forest models for the entire tropics, with variables ordered by their total predictive power and coloured by type: environmental variables (purple), variables concerning the surrounding forest landscape (green), variables relating to levels of human pressure (yellow), and variables relating to the location of the regenerating forest (blue). Random forest model error (RMSE) and variance explained (R^2) for each region is shown in the bottom corner of each panel. FLII refers to the 'Forest Landscape Integrity Index'[35].

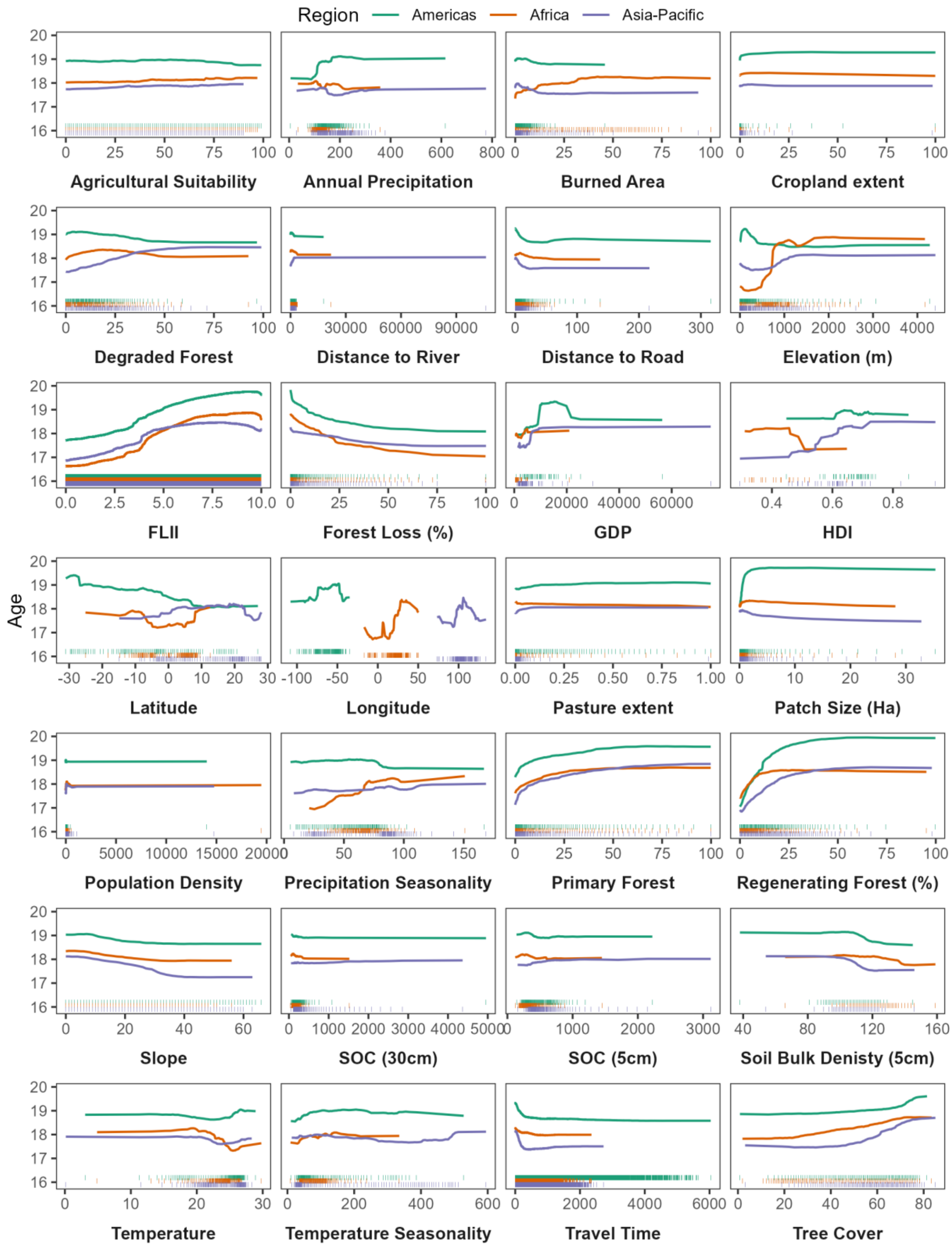

Supplementary Figure 4. Partial effects of the 28 continuous predictors of regenerating tropical moist forest age. Shown are partial effect plots on regenerating forest age across the three separate random forest regional models. The regional-level partial effects are coloured by region: Americas (green), Africa (orange), Asia-Pacific (purple). Distributions of data points for modelling partial effects in each region are shown at the bottom of each panel.

Supplementary Note 1- Data Comparison - Lesiv *et al.* (2022) global map of forest management

We also compared our map of regenerating tropical moist forest with a global map of forest management type for the year 2015[40]. This map was generated using a combination of GeoWiki based classifications of forest management types and machine learning to categorise the world's forests into management types at 100m resolution. We consulted the author's confidence matrix, and crossed the random sample of regenerating forest points used in the random forest modelling with Lesiv *et al.*'s map, and found good overall agreement. We found that only 8.2%, 9.1% and 22.7% of regenerating forests in our map were mapped as something other than natural forest (Plantations or Agroforestry) by Lesiv *et al.* (2015) in the Americas, Africa and Asia-Pacific respectively. However, the Lesiv *et al.*[40] map shows significant uncertainty in tropical regions, and when considering only pixels mapped with >50% confidence, the disagreements fall to 3.8%, 5.7% and 8.9%, respectively. In addition, the majority of disagreements with our naturally regenerating points were classified by Lesiv *et al.*[40] as 'agroforestry', which they define as also including areas of shifting cultivation. We would argue that such areas account for a large proportion of the younger regenerating forest mapped in this study, and this therefore does not represent a disagreement in actual land cover but is the result of variable definitions of regenerating forest.

Supplementary Table 2. Overlap of differentially mapped classes between this study and Lesiv *et al.* map of forest management.

Category	Region	Overall Overlap (%)	50% Confidence Overlap (%)	70% Confidence Overlap (%)
Plantation (Tree or Oil Palm)	Americas	1.8	1	0.4
Agroforestry	Americas	6.4	3.7	0.8
Plantation (Tree or Oil Palm)	Africa	0.2	0.1	<0.1
Agroforestry	Africa	8.9	5.6	1.2
Plantation (Tree or Oil Palm)	Asia-Pacific	9.7	4.4	1.4
Agroforestry	Asia-Pacific	13.0	4.5	0.5

Supplementary Note 2- Data Comparison – MapBiomas

To compare our map of regenerating tropical moist forest age with similar products, we conducted a country-level comparison for Brazil with the 'Regenerating Vegetation Age' module from Collection 9 of the MapBiomas dataset[44,45]. MapBiomas is a land-cover dataset developed for Brazil used widely in forest-related research[88,89], which includes a 'Regenerating Vegetation Age' Dataset. Since both our dataset and the MapBiomas data are Landsat-derived, we were able to directly compare the estimated age of regenerating forest pixels in our dataset and the MapBiomas data, as well as compare the estimated area of regenerating forest across Brazil.

We limited the analysis of the MapBiomas data to areas classified as 'Forest Formation' in the land cover class dataset. To compare differences in area of regenerating forest across the two datasets, we aggregated both datasets up to 15km^2 grids covering Brazil, and summed the total area of regenerating forest in each 15km^2 pixel. The two datasets showed moderate correlation in regenerating forest area across 15km^2 pixels (R^2 = 0.49) and good agreement on the general spatial patterns of regenerating forest distribution (Supplementary Figure 5a), though MapBiomas mapped a greater extent of regenerating forest (16.0 Mha) compared to our data (10.6 Mha). To determine the correlation of estimated age of regenerating forest pixels, we took a random sample of 200,000 regenerating forest pixels from our data, and extracted the estimated age for those pixels from the MapBiomas data. Again, we found moderate correlation in estimated age between the two datasets (R^2 = 0.37, Supplementary Figure 5b). Discrepancies between the two datasets are likely primarily down to differences in the regenerating forest definition between the two datasets, with MapBiomas taking a much broader definition of natural forest formations that differ based on which region of Brazil they are found, whereas our data follows one pan-tropical definition of 'moist forest' with >90% canopy cover, and requires a minimum of three years of moist forest cover to classify a pixel as forest regrowth[21].

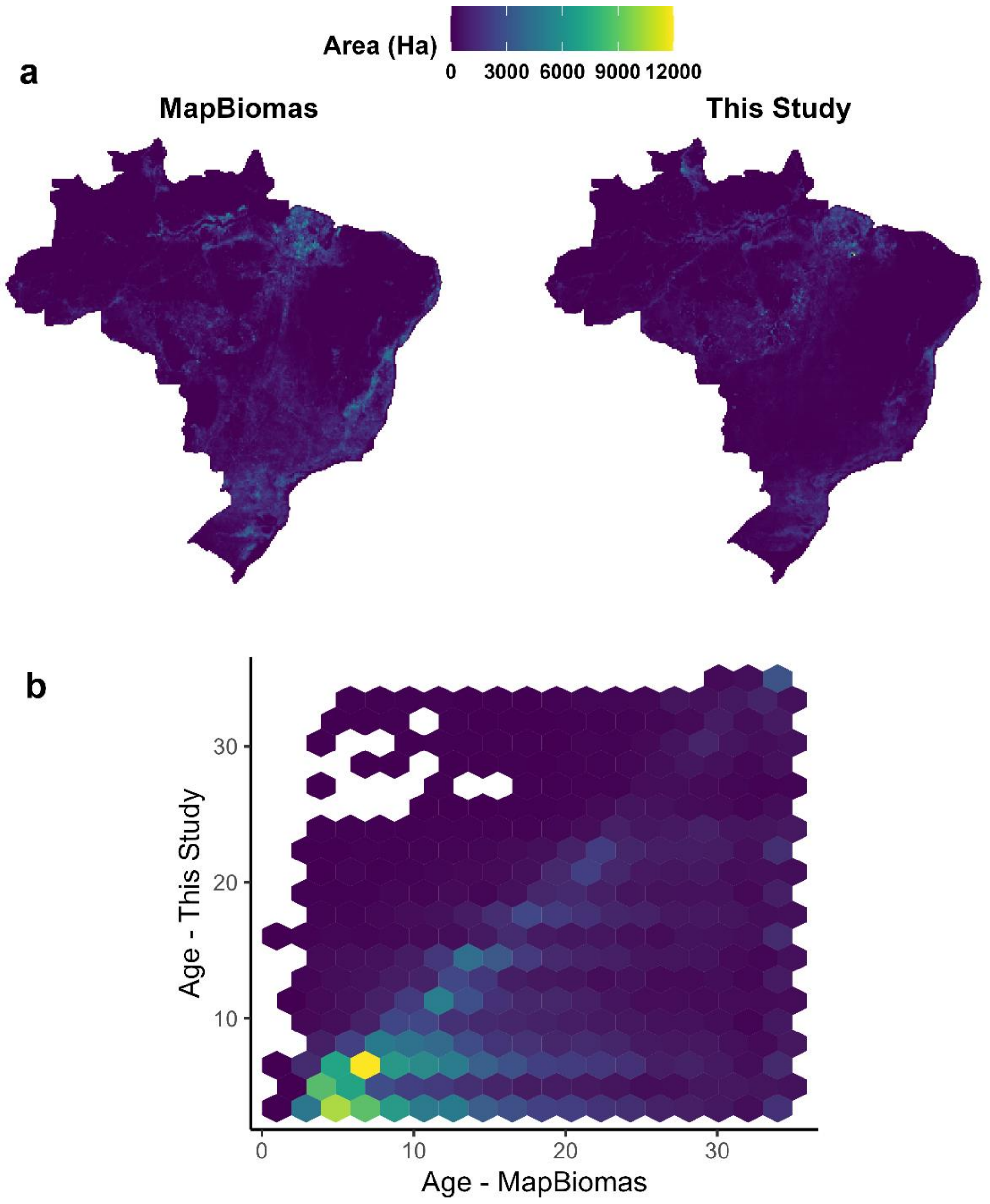

Supplementary Figure 5. Comparisons between the data in this study and MapBiomas. Shown are (a) the area of regenerating forest mapped by this study and MapBiomas at a resolution of 15km^2 (R^2 = 0.49), and (b) the correlation between age estimates from a random sample of 200,000 Brazilian regenerating forest points from this study and the age estimate from MapBiomas in the corresponding cell (R^2 = 0.37).

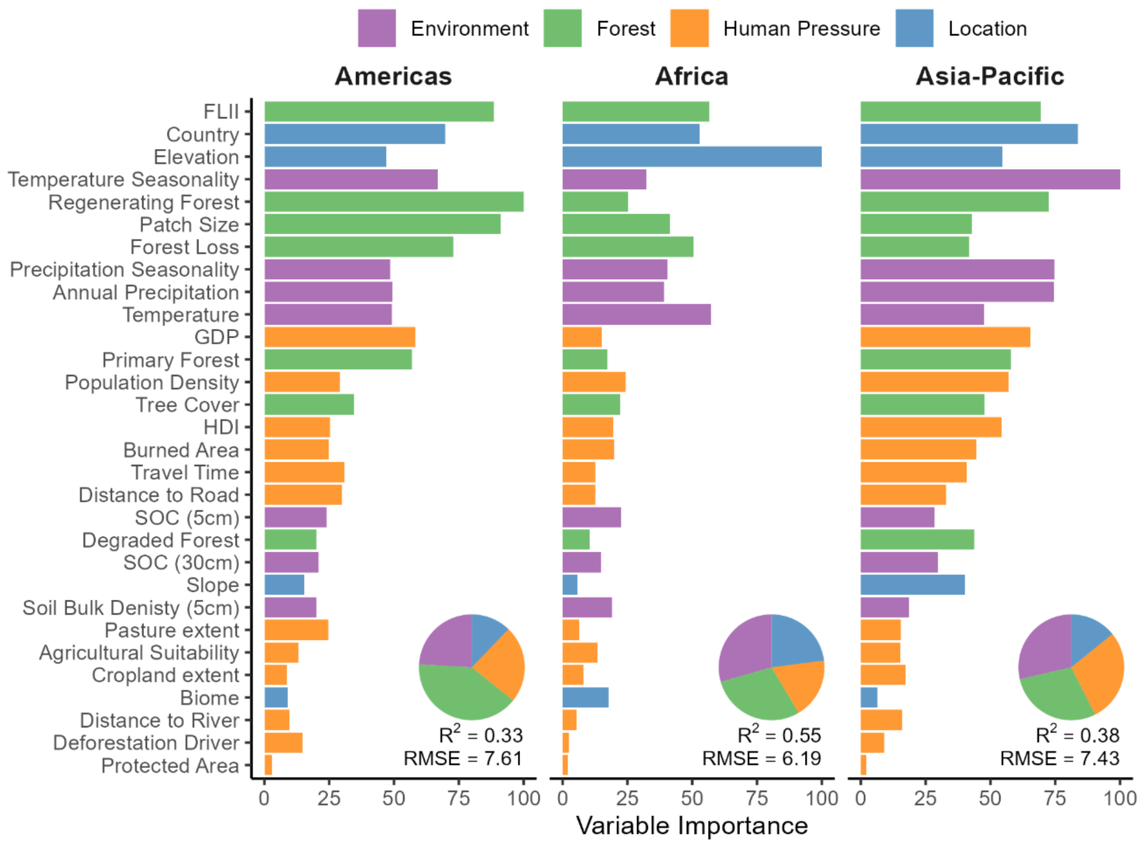

Supplementary Figure 6. Variable importance scores for predicting regenerating tropical moist forest age without longitude and latitude. Shown are the variable importance scores from the random forest models for each region, with variables ordered by their total predictive power across all three regional models and coloured by type: environmental variables (purple), variables concerning the surrounding forest landscape (green), variables relating to levels of human pressure (yellow), and variables relating to the location of the regenerating forest (blue). Random forest model error (RMSE) and variance explained (R^2) for each region is shown in the bottom corner of each panel. FLII refers to the 'Forest Landscape Integrity Index'[35].

Additional References

79. Hansen, M. C. *et al.* High-Resolution Global Maps of 21st-Century Forest Cover Change. *Science* **342**, 850–853 (2013).

80. Chen, Y. *et al.* Multi-decadal trends and variability in burned area from the fifth version of the Global Fire Emissions Database (GFED5). *Earth Syst. Sci. Data* **15**, 5227–5259 (2023).

81. Nelson, A. *et al.* A suite of global accessibility indicators. *Sci. Data* **6**, 266 (2019).

82. Kummu, M., Taka, M. & Guillaume, J. H. A. Gridded global datasets for Gross Domestic Product and Human Development Index over 1990–2015. *Sci. Data* **5**, 180004 (2018).

83. Ramankutty, N., Evan, A. T., Monfreda, C. & Foley, J. A. Farming the planet: 1. Geographic distribution of global agricultural lands in the year 2000. *Glob. Biogeochem. Cycles* **22**, 2007GB002952 (2008).

84. Zanaga, D. *et al.* ESA WorldCover 10 m 2021 v200. Zenodo https://doi.org/10.5281/ZENODO.7254221 (2022).

85. Zabel, F., Putzenlechner, B. & Mauser, W. Global Agricultural Land Resources – A High Resolution Suitability Evaluation and Its Perspectives until 2100 under Climate Change Conditions. *PLoS ONE* **9**, e107522 (2014).

86. Lehner, B. & Grill, G. Global river hydrography and network routing: baseline data and new approaches to study the world's large river systems. *Hydrol. Process.* **27**, 2171–2186 (2013).

87. Hanson, J. O. wdpar: Interface to the World Database on ProtectedAreas. *J. Open Source Softw.* **7**, 4594 (2022).

88. Heinrich, V. H. A. *et al.* Large carbon sink potential of secondary forests in the Brazilian Amazon to mitigate climate change. *Nat. Commun.* **12**, 1785 (2021).

89. Qin, Y. *et al.* Forest conservation in Indigenous territories and protected areas in the Brazilian Amazon. *Nat. Sustain.* **6**, 295–305 (2023).
