# Supplementary material for: The pan-tropical age distribution of regenerating tropical moist forest
Source: Nat Ecol Evol. 2025 May 20;9(7):1205–13. doi: 10.1038/s41559-025-02721-8 (PMC12240804; doi:10.1038/s41559-025-02721-8)
Supplement: Supplementary file 2 — Reporting Summary [file 41559_2025_2721_MOESM2_ESM.pdf]

nature portfolio

Corresponding author(s): Christopher Bousfield

Last updated by author(s): Apr 4, 2025

Reporting Summary

Nature Portfolio wishes to improve the reproducibility of the work that we publish. This form provides structure for consistency and transparency in reporting. For further information on Nature Portfolio policies, see our Editorial Policies and the Editorial Policy Checklist.

Statistics

For all statistical analyses, confirm that the following items are present in the figure legend, table legend, main text, or Methods section.

n/a	Confirmed	
☒	☐	The exact sample size (*n*) for each experimental group/condition, given as a discrete number and unit of measurement
☐	☒	A statement on whether measurements were taken from distinct samples or whether the same sample was measured repeatedly
☐	☒	The statistical test(s) used AND whether they are one- or two-sided<br>*Only common tests should be described solely by name; describe more complex techniques in the Methods section.*
☐	☒	A description of all covariates tested
☒	☐	A description of any assumptions or corrections, such as tests of normality and adjustment for multiple comparisons
☐	☒	A full description of the statistical parameters including central tendency (e.g. means) or other basic estimates (e.g. regression coefficient) AND variation (e.g. standard deviation) or associated estimates of uncertainty (e.g. confidence intervals)
☒	☐	For null hypothesis testing, the test statistic (e.g. *F*, *t*, *r*) with confidence intervals, effect sizes, degrees of freedom and *P* value noted<br>*Give P values as exact values whenever suitable.*
☒	☐	For Bayesian analysis, information on the choice of priors and Markov chain Monte Carlo settings
☒	☐	For hierarchical and complex designs, identification of the appropriate level for tests and full reporting of outcomes
☒	☐	Estimates of effect sizes (e.g. Cohen's *d*, Pearson's *r*), indicating how they were calculated

Our web collection on statistics for biologists contains articles on many of the points above.

Software and code

Policy information about availability of computer code

Data collection	The code used to generate the results are available at https://doi.org/10.5281/zenodo.1512087078<br><br>The analysis was done using a combination of Google Earth Engine and RStudio (4.4)
Data analysis	The code used to generate the results are available at https://doi.org/10.5281/zenodo.1512087078<br><br>The analysis was done using a combination of Google Earth Engine and RStudio (4.4)

For manuscripts utilizing custom algorithms or software that are central to the research but not yet described in published literature, software must be made available to editors and reviewers. We strongly encourage code deposition in a community repository (e.g. GitHub). See the Nature Portfolio guidelines for submitting code & software for further information.

Data

Policy information about availability of data

All manuscripts must include a data availability statement. This statement should provide the following information, where applicable:

- Accession codes, unique identifiers, or web links for publicly available datasets
- A description of any restrictions on data availability
- For clinical datasets or third party data, please ensure that the statement adheres to our policy

The underlying TMF dataset used in this study is freely accessible and available for download at https://forobs.jrc.ec.europa.eu/TMF/data. Additional data sources used for random forest modelling are also freely accessible and available for download in the links provided in the supplementary material. The final map of regenerating tropical moist forest age for the year 2023 is available for download at https://doi.org/10.5281/zenodo.1512087078

Research involving human participants, their data, or biological material

Policy information about studies with human participants or human data. See also policy information about sex, gender (identity/presentation), and sexual orientation and race, ethnicity and racism.

Reporting on sex and gender	NA
Reporting on race, ethnicity, or other socially relevant groupings	NA
Population characteristics	NA
Recruitment	NA
Ethics oversight	NA

Note that full information on the approval of the study protocol must also be provided in the manuscript.

Field-specific reporting

Please select the one below that is the best fit for your research. If you are not sure, read the appropriate sections before making your selection.

☐ Life sciences ☐ Behavioural & social sciences ☒ Ecological, evolutionary & environmental sciences

For a reference copy of the document with all sections, see nature.com/documents/nr-reporting-summary-flat.pdf

Ecological, evolutionary & environmental sciences study design

All studies must disclose on these points even when the disclosure is negative.

Study description	Open source remotely-sensed forest cover datasets were combined to produce a pan-tropical map of regenerating tropical forest age. Random forest models were also used to assess the importance of 32 predictors in predicting regenerating forest age.
Research sample	Overall dataset is based on all 2023 detections of 'regrowth forest' by the JRC TMF transition map. Sample for the random forest modeling is a randomly selected sample of 10,000 points for each potential age (3-34 years), which is aimed to represent the entire regenerating forest extent.
Sampling strategy	Random sample of 10,000 taken for each age group (3-34 years)
Data collection	NA
Timing and spatial scale	JRC TMF Annual change dataset covers the years 1990-2023, and covers the entire extent of tropical moist forest
Data exclusions	No data were excluded from the analysis
Reproducibility	Random forest models were rerun to check consistent fit
Randomization	Separate models were fit for each key tropical region (Americas, Africa, Asia-Pacific). Samples for models were taken randomly.
Blinding	Blinding was not relevant in this study.

Did the study involve field work? ☐ Yes ☒ No

Reporting for specific materials, systems and methods

We require information from authors about some types of materials, experimental systems and methods used in many studies. Here, indicate whether each material, system or method listed is relevant to your study. If you are not sure if a list item applies to your research, read the appropriate section before selecting a response.

Materials & experimental systems

n/a	Involved in the study
☒	☐ Antibodies
☒	☐ Eukaryotic cell lines
☒	☐ Palaeontology and archaeology
☒	☐ Animals and other organisms
☒	☐ Clinical data
☒	☐ Dual use research of concern
☒	☐ Plants

Methods

n/a	Involved in the study
☒	☐ ChIP-seq
☒	☐ Flow cytometry
☒	☐ MRI-based neuroimaging

Plants

Seed stocks	NA
Novel plant genotypes	NA
Authentication	NA
